# Infected bone resection plus adjuvant antibiotic-impregnated calcium sulfate versus infected bone resection alone in the treatment of diabetic forefoot osteomyelitis

**DOI:** 10.1186/s12891-019-2635-8

**Published:** 2019-05-24

**Authors:** Cheng-He Qin, Chun-Hao Zhou, Hui-Juan Song, Guo-Yun Cheng, Hong-An Zhang, Jia Fang, Rui Tao

**Affiliations:** 1 Department of Orthopaedics and Traumatology, Guangdong Second Provincial General Hospital, Guangzhou, 510317 People’s Republic of China; 2 Department of Orthopaedics and Traumatology, Provincial Key Laboratory of Bone and Cartilage Regenerative Medicine, Nanfang Hospital, Southern Medical University, Guangzhou, 510515 People’s Republic of China; 3 Department of Nursing, Nanfang Hospital, Southern Medical University, Guangzhou, 510515 People’s Republic of China

**Keywords:** Calcium sulfate, Diabetic foot osteomyelitis, Surgical treatment

## Abstract

**Background:**

Managing with diabetic foot osteomyelitis (DFO) is challenging. Even after infective bone resection and thorough debridement, DFO is still difficult to cure and has a high recurrence rate. This retrospective study aims to compare the outcomes of two treatment methods, infected bone resection combined with adjuvant antibiotic-impregnated calcium sulfate and infected bone resection alone, for the treatment of diabetic foot osteomyelitis.

**Methods:**

Between 2015 to 2017, 48 limbs (46 patients) with DFO met the criteria were included for assessment. 20 limbs (18 patients) were included in the calcium sulfate group (the CS group) in which vancomycin and/or gentamicin-impregnated calcium sulfate was used as an adjuvant after infected bone resection while 28 limbs (28 patients) as the control group were undergone infected bone resection only. Systemic antibiotics, postoperative wound care and offloading were continued to be applied following surgery in both groups. The time to healing, healing rate, recurrence rate and amputation rate were compared between the two groups.

**Results:**

In total, 90% (18/20) limbs in the CS group as compared to 78.6% (22/28) infected limbs in the control group went to heal (*P* = 0.513). The Mean time to healing was 13.3 weeks in the CS group and 11.2 weeks in control group (*P* = 0.132). Osteomyelitis recurrence rate was 0% (0/18) in the CS group and 36.4% (8/22) in the control group (*P* = 0.014). Postoperative leakage in calcium sulfate group was 30.0% (6/20) with a mean duration of 8.5 weeks. Amputation rate in the control group was 7.1% (2/28) compared to 0% (0/20) in the CS group (*P* = 0.153).

**Conclusions:**

Antibiotic-impregnated calcium sulfate as an adjuvant prevents the recurrence of DFO but cannot improve the healing rate, reduce the postoperative amputation rate or shorten the time to healing. Prolonged postoperative leakage as the most common complication can be managed with regular dressing.

**Level of Evidence:**

III, Retrospective Comparative Study.

## Background

Diabetic foot osteomyelitis (DFO) is a common complication of patients with diabetic foot infections. It was reported that nearly 20–60% of patients with diabetic foot might suffer from DFO [1–3]. However, DFO is a difficult-to-treat infection disease, as the treatment of DFO might include the management of chronic ulcers, necrotic soft tissues, gangrenes and of course, the infected bones. Although various treatment methods have been adopted, unfortunately, suffered from DFO still means a high rate of amputation and mortality [4].

Currently, the mainstay treatments for DFO consist of antimicrobial therapy alone or in combination with surgical intervention depending on the severity of disease [5]. In the case of mild infection, antibiotic administration alone for several weeks received promising results [5–7]. However, damaged peripheral vessels condition may make it difficult for parental antimicrobial therapy to achieve satisfying local effects due to insufficient penetration [8]. Moreover, the optimal duration of antibiotic therapy is still controversial with formal studies reported the antibiotic therapy duration varied from 6 weeks to more than 40 weeks [6, 9, 10]. The prolonged duration of antibiotic therapy is limited by the advent of antibiotic-resistant bacteria and potential drug-induced gastrointestinal, liver and kidney injury [11]. For patients with pus, sequestrum, gangrene or antibiotic-resistant bacterial infection [5, 12], surgery is the cornerstone to remove dead tissues and eliminate the infections. For the latest decades, necrotic bones and tissues resection instead of amputation has been widely accepted in treating with DFO, as it removes the infected bones while preserves the healthy bones to minimize the biomechanical changes. However, when carrying out infected bone resection, the completely negative resection margin is relatively difficult to be identified, which may lead to the residue of pathogens. Furthermore, the removal of infected bone sometimes causes the formation of dead space, which will be filled with hematoma soon and provide an environment for the growth of bacteria. Muscle flap used to be a method to obliterate the defects caused by debridement, but it is limited when managing with deeper defects and may disturb the bone healing [13].

Local antibiotic delivery system has been widely used as an adjuvant after the surgical treatment of osteomyelitis and achieved good results [14, 15]. Compared with intravenous route, the local antibiotic delivery has the advantages of more accurate positioning, higher local concentration, less side effects and longer duration. At the same time, it works as a bone substitute which fills the dead space caused by bone resection and reduces the incidence of reinfection. Polymethyl-methacrylate cement (PMMA) has acted as an antibiotic carrier to fill the defects caused by debridement since *Buchholz* successfully applied it in joint prosthesis. However, its non-biodegradable characteristics, the high temperature it produces and a second surgery for removal all limit its application on osteomyelitis especially in DFO [16].

Nowadays, biodegradable antibiotic-impregnated materials such as calcium sulfate, calcium phosphate, bioactive glasses and collagen are gradually applied as a substitute for PMMA in the management of osteomyelitis. All materials mentioned above have advantages of biocompatibility and drug compatibility. Among those substitutes, calcium sulfate is most frequently used materials since it enjoyed some eminent advantages. To begin with, the elution characteristic of loaded antibiotics are now clearly illustrated, an initial burst of antibiotics releasing in the first 24 h or 48 h produces antibiotics levels about hundreds to thousands times higher than minimum inhibitory concentration (MIC), then the calcium sulfate releases all antibiotics it loaded gradually at relative slow pace and complete resorption in several weeks. This ideal elution duration makes it more available than collagen (too short) and calcium phosphate (too long) to be a bone graft. Furthermore, it hardly produces the foreign body reaction and helps the formation of new bone. The complications of calcium sulfate are also acceptable, including postoperative drainage and transient hypercalcemia [17].

Previous studies had reported that satisfying outcomes could be received when using antibiotic-impregnated calcium sulfate as an adjuvant after surgical treatment of DFO. However, few comparative studies had been carried out to confirm those results. This retrospective study was designed to observe the outcomes of surgical treatment combined with adjuvant antibiotic-impregnated calcium sulfate versus surgical treatment alone in the treatment of DFO and to compare the differences of healing rate, time to healing, osteomyelitis recurrence rate and amputation rate between two groups.

## Methods

### Participants

This retrospective study focused on patients with DFO treated in our orthopedic department from January 2015 to June 2017. The main inclusion criteria were as follows: 1) patients with DFO underwent surgical bone resection alone or surgical bone resection combined with adjuvant antibiotic-impregnated calcium sulfate. 2) patients persisted to the follow-up and had been followed for at least 12 months. The main exclusion criteria included: 1) Patients received major amputation or non-surgical treatments. 2) Patients were diagnosed with severe peripheral arterial disease or severe infection according to IDSA. 3) Patients lost to follow up or the follow-up was less than 12 months. Finally, 46 patients with 48 infected limbs met the criteria were included in the study.

### Study design

Before admitting to our department for surgical treatment, 46 patients (48 limbs) with suspicious DFO (suspected by clinical presentation and the active X-ray, MRI or probe-to-bone test results [7] were sampled using percutaneous bone biopsy [18] in our diabetic foot unit for culturing and histology test. Preoperative antibiotic therapy was applied empirically after sampling in the first several days and tailored to culture and susceptibility findings. For patient with negative culture result but accompanied with the presentation of inflammation and positive of histology test, empirical antibiotics were adjusted according to the inflammatory markers. DFO are usually polymicrobial and *Staphylococcus aureus* has been proven as the most common pathogens in DFO [19, 20]. Thus, it is a necessity that empirical treatment of DFO should consist of antibiotics with activity against *S. aureus*.

Depending on the calcium sulfate applied or not, 46 patients (48 limbs) were divided into two groups: the CS group and the control group. The characteristics of patients in two groups were presented in Table 1. 20 limbs (18 patients) as the CS group were locally applied with vancomycin and/or gentamicin-impregnated calcium sulfate as an adjuvant after surgical bones resection while 28 limbs (28 patients) as the control group received surgical bones resection only. All surgical procedures were carried out by two experienced surgeons. The surgical treatment performed as the resection of infected bones and removal of the necrotic soft tissues. Healthy bones and soft tissues were preserved as far as possible for minimizing the biomechanical changes and covering the wounds. Once the infected tissues were completely removed, patients in two groups were treated with intravenous antibiotics in 2 weeks individually and followed by oral antibiotics for 4 weeks, according to the recommendation of the International Working Group of Diabetic Foot (IWGDF) [21, 22]. For postoperative wounds care, patients were suggested to offload in involved limbs. Routine dressings and skin moisturizers were applied every two days until wound healing or infection recurring. Once wounds achieving healing, patents were educated never walk in shoes that contributed to a foot ulcer. Customized insoles and shoes were recommended to reduce pressure transfer during follow-up.

**Table 1:** Preoperative characteristics of patients in two groups

	The CS group	The control group	*P* value
Infected limbs	20	28	–
Age (years)	59.2 (43–76)	61.8 (47–83)	0.353
Sex (Male)	9	17	0.416
Side (Left)	9	11	0.883
Mean duration of DFO (weeks)	15 (1–77)	17 (2–257)	0.804
The Texas classification system			
III B	17	21	0.631
III D	3	7	
Hypertension	8	15	0.465
Renal insufficiency	9	17	0.635
Mean ankle brachial index (ABI)	1.06 (0.82–1.43)	0.98 (0.65–1.17)	0.107
Mean WBC count (×10^9^/L)	8.09 (2.81–14.23)	8.03 (3.96–12.07)	0.936
Mean CRP (ng/L)	32.90 (1.17–165.47)	31.93 (1.20–114.40)	0.927
Mean ESR (mm/h)	86 (34–134)	88 (30–140)	0.829
Mean Albumin (g/L)	33.3 (20.9–42.6)	31.8 (24.7–36.9)	0.304
Mean Creatinine (μmol/L)	135 (38–682)	113 (33–513)	0.586
Mean HbA1c (%)	8.6 (5.6–10.1)	8.2 (4.8–11.2)	0.768

Abbreviations: *CS* Calcium sulfate, *DFO* Diabetic foot osteomyelitis, *WBC* white blood cell, *ESR* Erythrocyte sedimentation rate, *HBA1c* Glycosylated hemoglobin A1c

In this single-stage study, we defined the wound healing as complete epithelialization covered the wound and the absent of infection. Non-healing was defined if the wound was infected before healing and was treated with a second operation or antibiotics. Osteomyelitis recurrence was defined if the appearance of bone infection was presented at the same or adjacent site after wound healing. Patients suffered from non-healing were excluded from the further calculation for recurrence rate even if the wounds eventually healed with subsequent therapy.

### Operative technique

Surgical procedures were carried out after spinal, nerve block or regional anesthesia. In the CS group, necrotic granulation tissues, pus and infected soft tissues in the ulcers were removed until the bleeding tissue has been exposed. Following the removal of ulcers or sinus, the bone procedures were carried out. If osteomyelitis was located in diaphyses, devitalized bones in the base of ulcers were exposed and excised to the level of healthy cancellous and cortical bone. An extra 2 mm of healthy bone was also resected in the prophylaxis of residual pathogens. If possible, the bases of metatarsal and phalangeal bones were necessary to be preserved for healthy tendons attaching. When infections were located in interphalangeal or metatarsophalangeal joints, however, the joints as well as partial distal and proximal bones were needed to be excised. Fibrous tissues, fascia and tendons nearby were also completely removed in case the residue of pathogens. Following bones resection, the defects were irrigated with 0.05% chlorhexidine solution and sterile saline solution. If necessary, Kirschner wires were adopted to maintain the bones stable. After removal of infected bones and necrotic soft tissues, antibiotic-impregnated calcium sulfate was prepared. Vancomycin and/or gentamicin was mixed into the synthetic calcium sulfate (Stimulan, Biocomposite Ltd., UK) with a recommended ratio: 0.5 g vancomycin with 5 ml calcium sulfate or 80 mg gentamicin with 5 ml calcium sulfate. Then they were dissolved with sterile saline solution and injected into the dead space (range from 0.5 ml to 5 ml individually). After operations, the wounds were sutured primarily without tension. In the control group, patients received the same operation expect for the application of antibiotic-loaded calcium sulfate.

### Statistical analysis

Data were collated using Microsoft Excel (Redmond, Washington) and analyzed using SPSS v20 (SSPS Inc., Chicago, Illinois). Continuous variables which were verified of normal distribution and the homogeneity of variance were compared using Independent-Samples T Test; Continuous variables which failed to pass normality test were compared using a Mann–Whitney *U* test. Pearson χ2, Continuity Correction Chi-square Test or Fisher Exact Test were used in comparing the demographic data, healing rate, recurrence rate and amputation rate. *P* < 0.05 was considered statistically significant.

## Results

From 2015 to 2017, 46 patients (48 limbs) met the criteria were included in the study. The locations of DFO were 16 in phalanges (7 in the CS group), 13 in metatarsal bones (5 in the CS group) and 19 in both phalanges and metatarsal bones (8 in the CS group).

The preoperative culture results were presented in Table 2. 95.0% (19/20) samples in the CS group showed positive culture results with total of 24 bacterial spices isolated. In control group, 89.3% (25/28) samples were culture-positive with 40 isolated bacterial spices. 35.0% (7/20) infected limbs in the CS group were monomicrobial infections compared to 42.9% (12/28) monomicrobial infections in the control group. *Staphylococcus aureus* was the most common pathogen isolated by culture followed by *Escherichia coli* and *Enterococcus faecalis.*

**Table 2:** The microbiological findings in two groups

microbiological findings	The CS group	The control group
Monomicrobial infections	7 (35.0%)	12 (42.9%)
Total bacterial spices	24	39
*Staphylococcus aureus*	8	8
*MRSA*	1	0
*Escherichia coli*	2	8
*Enterococcus faecalis*	2	5
*Proteus species*	2	3
*Klebsiella species*	2	1
*Pseudomonas aeruginosa*	1	3
*Staphylococcus epidermidis*	1	0
*Streptococcus species*	2	2
*Acinetobacter baumannii*	0	2
*Candida albicans*	0	2
*Enterobacter cloacae*	0	1
*Stenotrophomonas maltophilia*	1	0
*Myroides odoratimimus*	1	0
*Negative finding*	1	4

Abbreviations: *CS* Calcium sulfate*, MASA Methicillin resistantstaphylococcus aureus*

**Fig. 1:**
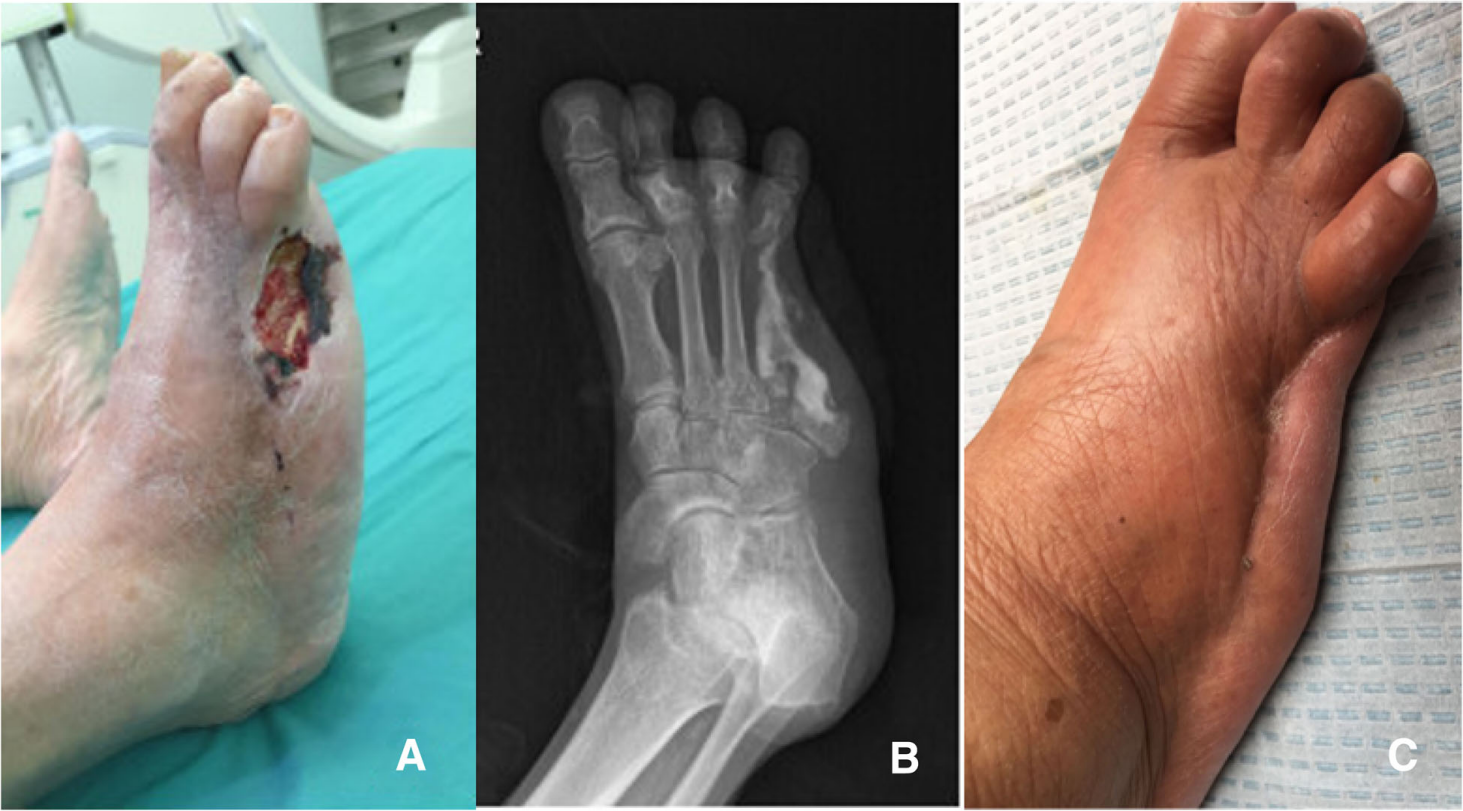
A patient with the fourth infected metatarsal (the fifth phalanx and metatarsal had been resected 5 years ago) were resected to the base of metatarsal before the vancomycin-impregnated calcium sulfate was injected into the dead space. **a** The presentation of wound before the operation. **b** The X-ray presentation 3 days after operation. **c** The ulcer had healed and no symptoms of osteomyelitis were presented 1 year after operation

**Fig. 2:**
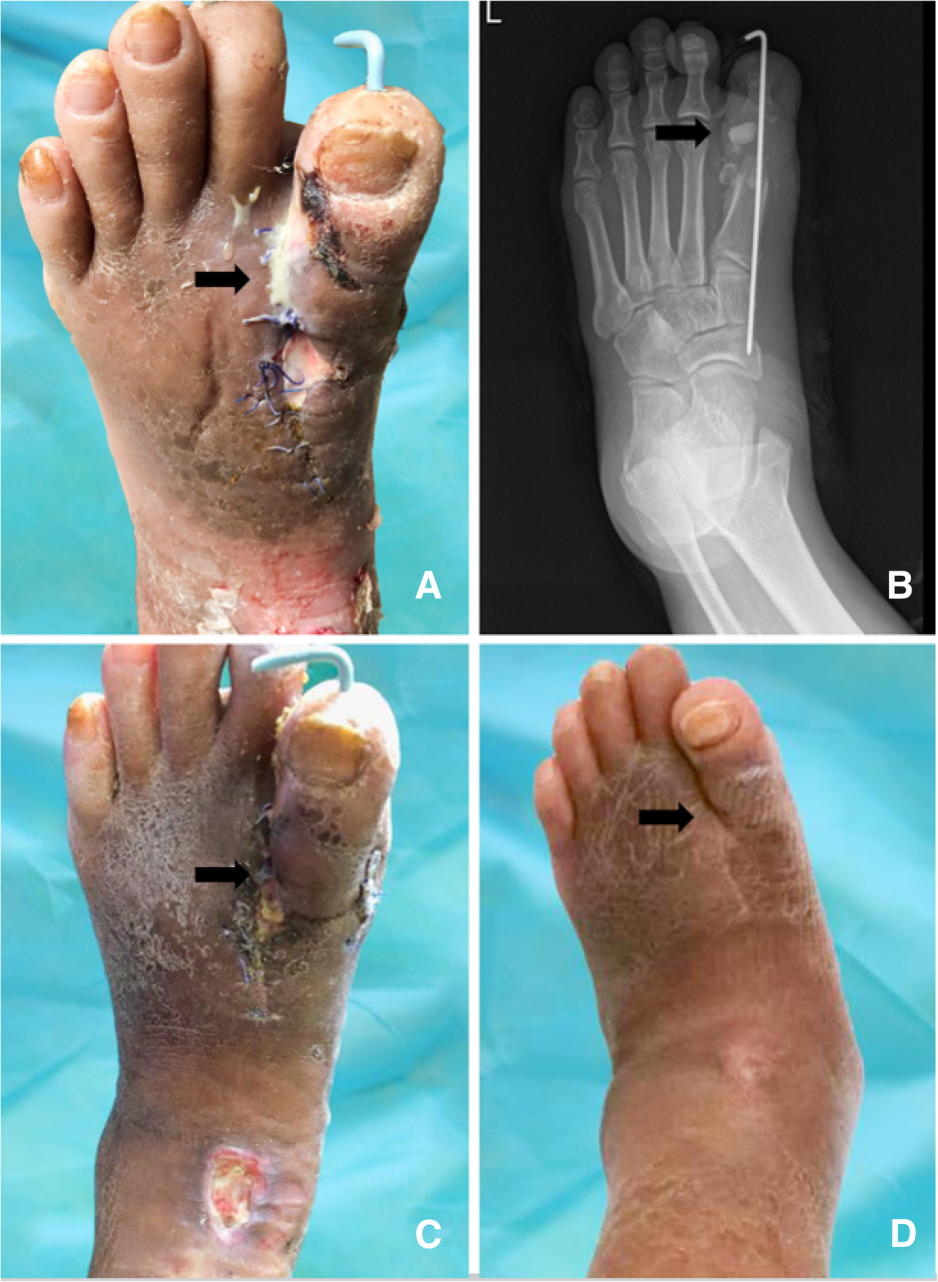
A 62-yaer-old patient with DFO on left first metatarsophalangeal joint. The metatarsophalangeal joint, partial metatarsal and phalange were removed. **a** The postoperative leakage of calcium sulfate. This sterile leakage was demonstrated as a kind of white, foamy, antibiotics-containing fluid. **b** X-ray presentation. Vancomycin-impregnated calcium sulfate lump was degrading. **c** 7 weeks after operation, the skin needed to be moistened. Superficial ulcer in ankle could be managed with dressing. **d** 1 year after surgical treatment. Although edema was presented, the operative wound healed and the symptoms of infection were disappeared

Twenty limbs (41.7%) as the CS group were applied with antibiotic calcium sulfate after infected bone resection compared to 28 limbs (58.3%) as the control group underwent infected bone resection alone. The follow-up outcomes of two groups were presented in Table 3. During follow up, 2 patients in the CS group died of cardiovascular disease after surgery, but the wounds had healed before death and did not recur within 1 year after surgery. 2 patients applied antibiotic-impregnated CS are presented in Figs. 1 and 2.

**Table 3:** The follow-up outcomes of two groups

	The CS group	The control group	*P* value
Mean hospital stay (days)	24.8 (7–59)	28.3 (9–56)	0.341
Mean follow-up duration(months)	17.6 (12–38)	20.1 (12–30)	0.120
Preoperative antibiotics duration (days)	13.5 (4–30)	15.6 (6–28)	0.142
Postoperative IV antibiotics duration (days)	8.8 (3–14)	9.8 (2–14)	0.569
Postoperative healing rate	90.0% (18/20)	78.6% (22/28)	0.513
Mean healing duration (weeks)	13.3 (5–30)	11.2 (2–26)	0.132
Recurrence rate	0.0% (0/18)	36.4% (8/22)	0.014
Postoperative leakage rate	30% (6/20)	–	–
Postoperative leakage duration (weeks)	8.5 (4–13)	–	–
Amputation rate	0.0% (0/20)	7.1% (2/28)	0.153

## Discussion

According to the severity of infections and local blood supply, diabetic foot osteomyelitis can be managed with conservative treatment or surgery. When accompanied with pus, substantial bone necrosis, gangrene, recurrent ulcer or antibiotic-resistant bacteria infection, surgery is recommended to remove necrosis tissues, reduce the antibiotic therapy duration and correct bone deformity to promote healing [23]. However, even surgical treatment also has its own limitations. Due to the bone removal, biomechanics in foot is inevitably changed, which may lead to the ulcer formation in a new position. Thus, postoperative offloading with customized insoles and shoes was essential to promote healing and prevent infection recurrence. Furthermore, during infected bone resection, the clear margin of bone and soft tissue is fairly difficult to identify. The exact extent for infected bone resection is largely depended on the intra-operative judgement of the surgeons and thus sometimes lead to the non-healing or recurrence of osteomyelitis. To eradicate the residual infection, local antibiotic-impregnated calcium sulfate was applied in our study as the high local antibiotic level it produced.

In total, 90.0% (18/20) limbs in CS group and 78.6% (22/28) limbs in control group healed after the first operation. The higher healing rate in CS group is in accordance with the retrospective study of *Rajesh M. Jogia* et al., who reported all DFO limbs (20 patients) achieved healing after surgical debridement combining with antibiotic-impregnated calcium sulfate beads application [24]. Similarly, *Noman Shakeel Niazia* et al. retrospectively studied 70 patients with DFO who received debridement and adjunctive antibiotic-loaded calcium sulfate treatment. During an average follow-up of 10 months, infection eradication and wound healing were achieved in 90 and 81% limbs respectively [25]. Proper explanation of the high healing rate is that much higher antibiotic concentration reached topically can eradicate more residual organisms with the resorption of the calcium sulfate. Former studies have shown antibiotic levels surpass 200 times the MIC [14] for organisms over days and still retain antimicrobial effect after 6 weeks to 3 months [26, 27], which is sufficient to penetrate the biofilm and eradicate the residual organisms. Unfortunately, a significant difference in the healing rate between the two groups was not found through statistical analysis (90% in CS group versus 78.6% in control group, *P*>0.05). Combining with the previously-reported efficacy of antibiotic-impregnated calcium sulfate in eradicating infections and well-controlled variables (similar blood supply condition, appropriate wound care and postoperative offloading between two groups) in our study, we deem that the small group of patients included may cause the absence of significant differences in the healing rate. This explains why the healing rates of two groups are not statistically significant. With regard to the recurrence rate, such high topical antibiotic concentration and long therapeutic duration explained that the much lower recurrence rate of antibiotic-impregnated calcium sulfate group than the control group. *Rajesh M. Jogia* et al. reported no recurrence in 20 patients who received surgical debridement combined with gentamicin or vancomycin-impregnated calcium sulfate beads within the follow-up of 12 months after surgical intervention, which is similar with the result in our study [26, 27].

However, our study failed to provide evidence that antibiotic-impregnated calcium sulfate will shorten the wound healing duration, which is different from the similar former study. *Martin Varga* et al. reported that application of gentamicin-impregnated collagen sponge shortened nearly 2 weeks of wound healing duration after minor amputation [28]. *Fabian G. Krause* et al. found that time to a dry wound was 5*.*2 weeks in the antibiotic-impregnated beads group and 7*.*0 weeks in the control group, even if no significance in two groups [29]. In this study, however, the mean duration of healing in CS group was 2.1 weeks longer than the mean healing duration in control group. Actually, we hypothesize that the prolonged leakage in CS group may interfere with the wound healing, but no previous study was found to support this hypothesis.

Prolonged postoperative leakage was found to be the most common complication in calcium sulfate treated patients. 30.0% limbs (6/20) suffered from prolonged postoperative leakage in CS group with a mean duration of 8.5 weeks. With regular dressing, all wounds achieved healing eventually. The drainage rate is similar with the former studies about chronic osteomyelitis and varies from 4.2 to 32% [13, 17, 30]. Other studies have reported the prolonged postoperative leakage in treating chronic osteomyelitis after using antibiotic-impregnated calcium sulfate, but achieving healing with appropriate wound care [13, 31]. In fact, prolonged postoperative leakage itself is neither an indication for a second surgery nor did it relate to reinfection of the wound [31]. Regularly dressing in outpatient is enough in dealing with the postoperative leakage. Vacuum-assisted Closure (VAC) was not used because we deemed that it might lower the topical antibiotic level when pumping the drainage. During surgical treatment, good soft tissue coverage and primary closure are essential methods in the prevention of postoperative leakage.

Severe side effects were not found excluding postoperative leakage in CS group. The explanation is that the dose of vancomycin or gentamicin administered locally was less than 0.5 g (vancomycin) or 100 mg (gentamicin), which means the systemic concentration reached was much lower than the same dose applied intravenously because of the slow releasing of antibiotics with the degradation of calcium sulfate. Unfortunately, systemic drug concentration was not obtained to confirm our hypothesis. In their study, *Zhang* et al. measured the blood vancomycin levels in 24 osteomyelitis patients locally applied with vancomycin-impregnated calcium sulfate beads (range from 1.5 ml to 5 ml with a ratio of 1 g vancomycin:5 ml calcium sulfate). The results showed that the mean blood vancomycin level was still within a safe range for application [32]. *P. Wahl* et al. found that even 6 g vancomycin was applied locally, the systemic concentration remained within a safe range and local concentration was still below the reported cell toxicity thresholds [27].

To our knowledge, our study is the first retrospective comparative study comparing the outcomes of infected bone resection combined with adjunctive antibiotic-impregnated calcium sulfate versus infected bone resection in the treatment of DFO. The limitations of our study are mainly in two aspects. Firstly, the follow-up duration in two groups may not be enough to show the outcomes of all patients, which may influence the healing rate, recurrence rate and amputation rate in our study. Furthermore, it is a retrospective study for forefoot DFO with a small group of patients, the additional studies are necessary to confirm our findings.

## Conclusion

Application of the antibiotic-impregnated calcium sulfate as an adjuvant can be regarded as efficacious for preventing the recurrence of forefoot DFO. However, evidence is not found that the use of antibiotic-impregnated calcium sulfate improves the healing rate, shorten the healing duration or reduce the amputation rate. Prolonged postoperative leakage as a common complication can be dealt with regular dressing.

## Abbreviations

CRP: C-reactive Protein
CS: Calcium Sulfate
DFO: Diabetic Forefoot Osteomyelitis
ESR: Erythrocyte Sedimentation Rate
IDSA: Infectious Diseases Society of America
IWGDF: International Working Group of Diabetic Foot
MIC: Minimum Inhibitory Concentration
MRI: Magnetic Resonance Imaging
PMMA: Polymethyl-methacrylate cement
VAC: Vacuum-assisted Closure
